# Using a Co-Creational Approach to Develop, Implement and Evaluate an Intervention to Promote Physical Activity in Adolescent Girls from Vocational and Technical Schools: A Case Control Study

**DOI:** 10.3390/ijerph14080862

**Published:** 2017-08-01

**Authors:** Maïté Verloigne, Teatske Maria Altenburg, Mai Jeanette Maidy Chinapaw, Sebastien Chastin, Greet Cardon, Ilse De Bourdeaudhuij

**Affiliations:** 1 Research Foundation Flanders, Egmontstraat 1, 1000 Brussel, Belgium; 2 Department of Movement and Sports Sciences, Faculty of Medicine and Health Sciences, Ghent University, Watersportlaan 2, 9000 Ghent, Belgium; sebastien.chastin@gcu.ac.uk (S.C.); greet.cardon@ugent.be (G.C.); ilse.debourdeaudhuij@ugent.be (I.D.B.); 3 Department of Public and Occupational Health, EMGO Institute for Health and Care Research, VU University Medical Center, van der Boechorststraat 7, 1081 BT Amsterdam, The Netherlands; t.altenburg@vumc.nl (T.M.A.); m.chinapaw@vumc.nl (M.J.M.C.); 4 Institute of Applied Health Research, School of Health and Life Sciences, Glasgow Caledonian University, Glasgow G4 0BA, UK

**Keywords:** female, participatory, sports participation, intervention, school

## Abstract

*Background:* As physical inactivity is particularly prevalent amongst lower-educated adolescent girls, interventions are needed. Using a co-creational approach increases their engagement and might be effective. This study aimed to: (1) describe the co-creation process, (2) evaluate how girls experienced co-creation, and (3) evaluate the effect of the co-creational interventions on physical activity, individual, sociocultural and school-based factors. *Methods:* Three intervention schools (n = 91) and three control schools (n = 105) across Flanders participated. A questionnaire was completed pre (September–October 2014) and post (April–May 2015). In between, sessions with a co-creation group were organised to develop and implement the intervention in each intervention school. Focus groups were conducted to evaluate the co-creational process. *Results:* School 1 organised sport sessions for girls, school 2 organised a fitness activity and set up a Facebook page, school 3 organised a lunch walk. Girls were positive about having a voice in developing an intervention. No significant effects were found, except for small effects on extracurricular sports participation and self-efficacy. *Conclusions:* Using a co-creational approach amongst adolescent girls might be a feasible approach. However, as interventions were minimal, effects were limited or undetectable. Future co-creation projects could consider the most optimal co-creation process, evaluation design and intensively test this approach.

## 1. Introduction

Physical activity is associated with improved physical and mental health among children and adolescents [1,2,3]. However, large proportions of children and adolescents fail to achieve the recommended 60 minutes of moderate-to-vigorous physical activity per day [4,5,6]. Adolescent girls especially have low levels of physical activity: during the transition from childhood to adolescence they have a steeper decline in physical activity and are therefore far less active than boys of the same age group [3,7]. A previous study using objective measures indeed showed that more than 90% of Flemish adolescent girls were not sufficiently active [8]. This lack of sufficient physical activity in adolescent girls is observed worldwide [9], so adolescent girls have been identified as a key target population for physical activity promotion [10,11,12]. To address this, several physical activity interventions have already targeted this specific group. A systematic review summarised the available evidence about physical activity interventions that targeted girls aged 5–18 years and found mixed results regarding effectiveness [13]. A meta-analysis focusing specifically on adolescent girls between 12 and 18 years showed a small but significant increase in physical activity outcomes [12]. It was concluded that school-based, multi-component interventions focusing on girls only seem to be most promising [12,13], but should involve adolescent girls in the planning of such interventions [13]. Indeed, only a few school-based interventions have engaged the target group in the development process of the intervention, although this would optimise the intervention [14,15]. Currently, most studies use a top-down approach where adolescents’ involvement is directed by researchers defining specific points where input is needed, such as providing feedback on specific intervention components that have been developed by researchers [16]. Using a co-creational approach as a participatory technique in which the target group is actively involved in the development and implementation of actual intervention strategies for a specific setting is a promising approach to increase engagement of the target group [17]. It might be a crucial step forward to work on physical activity promotion with the adolescent girls, as this group has a strong feeling of autonomy regarding health behaviour [18]. This co-creational approach fits within the principles of participatory health research. In the literature, different models of participatory health research have been described, basically sharing the same principles [19]. The most important principle is that the research is “participatory”, implying that the persons whose life or work is the subject of the research need to actively take part in the entire research process. In other words, there is a strong collaboration between researchers and the target group. Advantages of this approach include that it is pragmatic, local and tailored to the group of interest and specific setting for which it is created, resulting in contextually appropriate intervention strategies [18,20,21,22]. Further, it enhances co-learning, empowerment and ownership among the target group [22,23]. Finally, such approach is especially valuable to learn about and work with vulnerable, disadvantages or at-risk populations [24,25]. The focus of this study was on adolescent girls having a lower educational level, as they are even more at risk of engaging in unhealthy behaviours than their peers with a higher educational level [26,27,28], so they will especially benefit from this approach [25]. There is a considerable amount of participatory health research studies in the broader public health area, for example to improve quality of health services, to improve health in specific communities, or to remove threats to health in disadvantaged populations [29,30,31,32]. Recently, there has been increased attention for this approach in intervention studies to promote physical activity among the general population as well [14,20] because of the potential to reduce the gap between theory, research and practice.

The goal of our project was to develop, implement and evaluate physical activity-promoting interventions in co-creation with lower-educated adolescent girls in three secondary schools. This resulted in three main research questions, which were addressed by a mixed research design. The first aim was to describe the development, implementation and quantitative process evaluation of each co-created school-specific intervention to promote physical activity in lower-educated adolescent girls. The specific interventions were developed by a co-creation group of a researcher and adolescent girls from each of the three schools. The second aim was to qualitatively evaluate how these girls experienced being part of the co-creation group and how they perceived their own developed intervention, using focus groups. The third aim was to evaluate the effect of the co-creational interventions on girls’ physical activity and related individual, sociocultural and school-based factors in a case controlled trial by comparing intervention schools using a co-creational approach with control schools using a pre-test post-test design.

## 2. Materials and Methods

### 2.1. Recruitment of Schools and Participants

Convenience sampling was used to invite secondary schools that provided vocational or technical education (East-Flanders, Flanders, Belgium) to participate in the study. The reason to focus on vocational and technical education is because adolescents attending these type of schools are more likely to engage in unhealthy behaviours [26,27,28]. First, we sent a recruitment email to principals from six schools to act as an intervention school in our study, of which three agreed to participate. This number was considered feasible to apply the co-creational approach. Next, four schools were approached to act as a control school, of which three agreed to participate. The purpose was to invite all girls from the 10th Grade (including the majority of girls who were 16 years old) from both intervention and control schools to participate in the study (i.e., data collection at pre and post). Specifically for the intervention schools, the adolescent girls of the selected grades/classes were also invited to participate in a co-creation group at school. The selection of grades/classes was slightly different between schools (which will be presented in the Results), providing the opportunity to test the ability of co-creation in different contexts in order to provide a tailored and ecologically valid solution.

### 2.2. Overall Study Procedure

**Figure 1:**
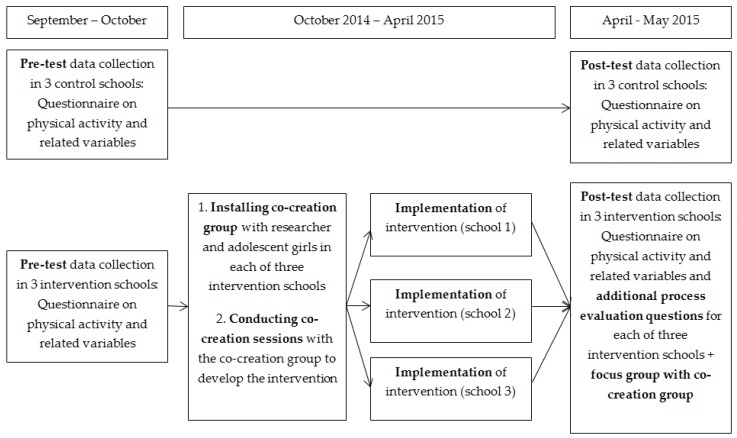
Overall study procedure of the co-creational project.

Between a pre-test (September–October 2014) and post-test (April–May 2015), a co-creation group (adolescent girls and a researcher) was installed in each intervention school. We did not set up a maximum number of girls in advance that could be part of the co-creation group, but we decided to await the number of subscriptions to see how we could optimally organise the co-creation group. Co-creation sessions were organised during lunch break in order to develop the intervention, which was subsequently implemented at each school. All girls participating in the co-creation group signed an informed consent. The three control schools did not receive any information on physical activity or health outside the normal curriculum. Figure 1 provides a detailed overview of the overall study procedure. The study protocol was conducted in accordance with the Declaration of Helsinki and was approved by the ethics committee of the Ghent University Hospital (B670201420580). The trial has been registered at ClinicalTrials.gov (NCT03135223; retrospectively registered on 18 April 2017).

### 2.3. Co-Creational Development of the School-Specific Interventions

Each intervention school followed the same process in developing their intervention. As a consequence of this approach, detailed descriptions of the development of the interventions were unknown before the start of the project. The school-specific intervention details are described in the results. First, a co-creation group was formed with the adolescent girls who gave consent to participate in the co-creation group and a university researcher. Throughout the school year (October 2014–February 2015), several co-creation sessions (six sessions in school 1; five sessions in school 2; eight sessions in school 3) were held during lunch break (about 50 min). In the first session, the researcher explained the aim of the project: the co-creation group could develop an intervention to promote physical activity among adolescent girls. It was emphasised that girls could choose which specific physical activity behaviour they wanted to change (e.g., sports participation, active transportation, daily physical activity, stair use, etc.), how they wanted to change it and that there was an available budget of maximum 500 euro to actualise their idea. The researcher also made clear that everyone in the co-creation group has equal standing (i.e., the principle of ownership in the co-creation process). It is important that the researcher in this process does not consider his or her academic knowledge or expertise as superior to the real-life knowledge and ideas of the girls. Next, the researcher asked the girls to think about how girls can be physically active in order to discuss different sub-behaviours of physical activity and asked which barriers and facilitators they perceived for engaging in these sub-behaviours. In each intervention school, the group brainstormed on what they could do to change those specific behaviours and ascertain what might be relevant for the girls in their school. At the end of this first co-creation session, a first proposal of a possible intervention was produced to promote physical activity that was contextually appropriate and viable. The following sessions focused on designing and making the intervention concrete, indicating that the content of the sessions depended on the chosen intervention (for example, practical organisation, communication and marketing, presenting the idea to school staff members, etc.). All decisions were jointly made by the girls and researcher. The researcher guided the girls through the development process of the intervention to ensure that the intervention was evidence-based, practical, realistic, safe, and so on, and helped the girls and school with implementing the intervention. To be capable of fulfilling this role, the researcher owned a pedagogical degree and followed workshops on Participatory Action Research.

### 2.4. Process Evaluation of the Co-Creation

To evaluate the process of co-creation and the co-created interventions, both quantitative and qualitative data were gathered to increase the validity of our findings. Quantitative data (i.e., questionnaire data) are numeric and measurable, whereas qualitative data (i.e., focus groups) can provide in-depth information on participants’ perceptions and experiences. For the quantitative part of the process evaluation in this project, process evaluation questions were added to the post-test questionnaire for each intervention school. In addition, we contacted the responsible school staff member of every intervention school during the following school year (2015–2016) to ask if they were willing to complete a short follow-up process evaluation questionnaire. For the qualitative part of the process evaluation, during the post-test at each intervention school a focus group was conducted with the co-creation groups of each school to evaluate the process of being part of the co-creation group and to evaluate their own developed intervention. 

### 2.5. Process Evaluation Measures

#### 2.5.1. Questions for Adolescents Girls from Intervention Schools

The process evaluation questions added to the post-test questionnaire were adapted to the intervention developed in each school, but generally included the same themes: participation in the intervention, satisfaction about the intervention, future organisation of the intervention, and if the intervention had prompted them to be more active. All questions are presented together with the results in the Results section. 

#### 2.5.2. Questions for School Staff from Intervention Schools

Nine months after post data collection (February 2016), one school staff member of every intervention school completed a short follow-up questionnaire regarding their view on the intervention developed and co-creation group. In addition, we asked if the school continued with the intervention or the co-creation group. All questions are presented together with the results in the Results section.

#### 2.5.3. Focus Group Protocol for Adolescent Girls from the Co-Creation Groups

The three focus groups were audiotaped and transcribed to facilitate analysis. The interview guide consisted of open-ended questions addressing the following topics: (1) reasons to participate in the co-creation group, (2) satisfaction with the progress of the co-creation group (e.g., practical organisation, inclusion of girls only, etc.), (3) satisfaction with the developed intervention, (4) expectations of participating in the co-creation group and (5) suggestions for the future. It was emphasised that negative experiences are useful too. Focus groups lasted about 30 min and were led by the same researcher who was present during the co-creation sessions to ensure that the girls would feel comfortable. 

### 2.6. Effect Evaluation of the Co-Creation

The adolescent girls who agreed to participate in data collection were asked to complete a questionnaire on physical activity and related individual, sociocultural and school-based factors at both pre-test and post-test. At the beginning of the questionnaire, the adolescent girls were informed that consent was automatically obtained when they voluntarily completed the questionnaire. 

### 2.7. Effect Evaluation Measures

#### 2.7.1. Physical Activity

The Flemish Physical Activity Questionnaire, which has been found reliable (Intraclass correlation coefficient of more than 0.70) and valid (Pearson correlation coefficients between 0.48 and 0.78 with accelerometers) among adolescents [33], was used to determine time spent in physical activity. Girls’ total physical activity (expressed in average minutes per day) was calculated by summing active transportation (i.e., minutes spent in active transportation to school and in leisure time per day) and sport participation (i.e., minutes spent in physical education, extra-curricular sports or physical activities at school and during leisure time per day).

#### 2.7.2. Individual, Sociocultural and School-Based Variables

Three individual, six sociocultural and three school-based variables were assessed. The individual and sociocultural variables were assessed using questions from previous studies examining health behaviour in adolescents [34,35,36], of which the predictive validity and reliability have been demonstrated previously [37,38]. The school-related variables were assessed using items from the Flemish Health Behaviour in School-aged Children questionnaire [39]. Individual variables included self-efficacy to be active, perceived benefits of physical activity and perceived barriers to be physically active. The mean values of four, six and five items, were used in this study to measure self-efficacy (Cronbach’s alpha = 0.85), perceived benefits (Cronbach’s alpha = 0.77) and perceived barriers (Cronbach’s alpha = 0.75) respectively. Single items were used to assess sociocultural variables, including peer and parental modelling of physical activity, peer and parental co-participation in physical activity and peer and parental encouragement to be physically active. School-based variables included relationship with classmates, involvement in organising school activities and relationship with teachers. The mean value of three items was used in this study to assess relationship with classmates (Cronbach’s alpha = 0.85). For all variables, a higher value represents a higher level (e.g., a higher mean value for perceived benefits of physical activity represents a stronger perception of benefits). All questionnaire items and response categories are displayed in Table 1. The reason to include these specific variables was the significant association with girls’ physical activity behaviour [40] and/or the hypothesis that co-creation could have an effect on these variables (e.g., on the relationship with classmates).

### 2.8. Analyses

To describe the co-creation of the intervention in each school and the quantitative process evaluation data for each intervention school (the first study aim), frequencies and descriptive statistics (using SPSS 23.0, IBM, Armonk, NY, USA) were used. To qualitatively evaluate how girls experienced being part of the co-creation group in the three intervention schools (the second study aim), data were thematically analysed via NVivo 11.0 (QSR International Pty Ltd., Doncaster, Australia) in several phases [41]. First, a coding scheme was developed based on the focus group protocol, consisting of the five main topics. Secondly, a combination of both axial coding and inductive coding was used in order to add other sub-themes that arose in the transcripts to the final coding template [41]. The final coding scheme was then used to code all transcripts. All codes of the transcripts were compared between co-creation groups for similarities and variability and interpreted [41]. To ensure the reliability of coding and data interpretations, two researchers independently carried out these analyses. Levels of physical activity and related individual, sociocultural and school-related factors at baseline and at post-test were reported for each intervention schools. To assess and quantify the effect of the physical activity interventions (study aim three), multilevel repeated measures analysis was performed using MLwiN 2.31 (Centre for Multilevel Modelling, University of Bristol, Bristol, UK). Multilevel modelling (three-level: measurement-pupil-school) was used to take clustering of two measurements of adolescent girls in schools into account (for a more complete analysis). Three skewed variables (active transportation to school, extracurricular sports participation, and sports participation in leisure-time) were log-transformed for analyses to improve normality. For ease of interpretation, non-transformed mean values are reported in the tables. Girls’ age was included as a covariate as the age range went up to 20.4 years (due to the selection of all grades in school 3 and a rather high prevalence of repeaters in vocational and technical education). The reported *β*-value for the interaction effect between “time” and “condition” can be interpreted as the difference in the change in outcome going from pre-test to post-test according to the condition to which children belong (intervention vs. control condition) *p*-values < 0.05 were considered statistically significant. The dataset has been included as Supplementary File 1.

## 3. Results

### 3.1. Description of Participating Schools

In four out of six schools (three control schools (n = 122) and one intervention school, School 2 (n = 76)), all adolescent girls from the 10th Grade were invited to participate in the study. However, in one intervention school (i.e., School 1), the principal selected one class from the 10th Grade (n = 14), as the other classes only or mostly contained boys. In another intervention school (i.e., School 3), the principal chose to involve all girls from the school (n = 34). This last school is a small school, offering part-time vocational education (combined with part time work) to pupils between 15 and 25 years and not operating with different grades, suggesting it was more practicable to invite all girls. In total, 27 classes across six schools participated in the data collection. For the first two research questions (See Section 3.2 and Section 3.3), only data from (girls in) intervention schools were used. For the third research question (See Section 3.4), data from girls in both intervention and control schools were used.

### 3.2. Co-Creational Development and Implementation of the School-Specific Physical Activity Interventions and Quantitative Process Analysis

General information about each intervention school, the co-creation group, the intervention developed and the implementation of the intervention are presented in Table 2. The quantitative process evaluation based on girls’ questionnaire data is presented in Table 3. The quantitative process evaluation based on the questionnaire data from school staff is presented in Table 4. The development of school-specific interventions is described below.

School 1: The girls from this co-creation group (n = 8; mean age 15.9 (0.7) years) indicated that many girls at the school did not have the resources or opportunities to participate in sports during leisure time and that there were at that time no opportunities to be physically active during lunch break. Therefore, the co-creation group decided to organise sport sessions during lunch break at school. As the group believed that they would need permission and help to organise their idea, they invited the physical education teacher to the next co-creation session. He gave permission to organise the sport sessions at the gymnasium and the organisation of some practical issues was discussed (e.g., availability of the gymnasium). During the next co-creation sessions, the group decided that the sessions would only be accessible for girls and not for boys in order to generate a comfortable feeling. The co-creation group chose four sports activities that were usually not offered in physical education and that are attractive for adolescent girls, namely (a) Zumba, (b) hip hop dance, (c) kickbox for girls with music and (d) girls’ work-out (i.e., strength training for girls). To announce the sport sessions, the girls designed a flyer/poster together with the researcher during one of the sessions. The girls and the researcher hung up the poster in the entrance hall of the school and the girls handed out flyers in the classes. The physical education teacher also announced the session on the digital school platform for pupils. The budget was used to make the poster and flyers and to pay for specialised sports teachers (who were approached by the researcher).

There were on average eleven girls present per sport session who informally gave feedback after each session that they enjoyed it and would participate in the next sessions as well. Only three girls from the co-creation group participated in all four sport sessions (reasons for not participating were cited in the focus groups and will be discussed further) and completed the process evaluation questionnaire. The physical education teacher who completed the follow-up process evaluation questionnaire for school staff was generally positive about the intervention and the co-creation group, but reported that both did not continue in the following school year. 

School 2: The girls in this co-creation group (n = 13; mean age 16.1 (0.6) years) indicated in the first session that girls of their age have different interests (for example, some like dancing, others like running, others like to do physical activities at home) and different time schedules (for example, some girls have time to be active immediately after school, other girls only during weekends). Because of these differences, the co-creation group decided to set up a Facebook page where they could post pictures and movies of exercises to do at home, healthy recipes, tips to be active, and so on. This Facebook page was set up in one of the co-creation sessions on the researcher’s laptop. Besides this Facebook page, the group also wanted to organise a single fitness activity for all pupils of Grade 10 (both boys and girls) during school hours, in which each individual would be free to choose the exercise they want to do. During the co-creation sessions, several practical issues were discussed: for example, it was decided to walk to the fitness centre from school as an additional physical activity and that every participant should receive a bottle of water and a piece of fruit, and a flyer with the link to the Facebook page in order to promote the page and to make clear that the fitness activity was an initiative from the co-creation group. The girls designed the flyer in one of the sessions together with the researcher. Further, the researcher suggested that it would be necessary to involve the school staff, so the co-creation group invited the principal and one of the school staff members to be present during one of the sessions to give their consent and to discuss practical issues: Grade 10 was divided into four groups; two groups each went to a fitness center during the morning school hours and two groups each went to a fitness center during the afternoon school hours. Every group was supervised by at least two school teachers who were informed by the principal and the school staff member involved. The budget was used to buy the fruit and water, to print the flyers, and to pay for the fitness activity of every adolescent.

We had no official records on how many of the 103 adolescents from Grade 10 were actually present during the fitness activity (for example, some adolescents were absent that day, some were not allowed to participate as a punishment for bad behaviour), however, 79% of the girls that completed the post-test questionnaire indicated that they participated in the fitness activity. Regarding the Facebook page, it was observed that most posts were placed by the researcher (in order to motivate others to post something as well), and only a limited amount of posts by the girls from the co-creation group, despite the fact that both parties were “owners” of the page. Posts included examples of abdominal exercises to do at home, motivational quotes to be physically active or to do sports, healthy recipes, and so on. The school coordinator who completed the follow-up process evaluation questionnaire was generally positive about the intervention developed and the co-creation group, but reported that both did not continue in the following school year. 

School 3: The girls in this co-creation group (n = 2; mean age 17.2 (0.0) years) indicated that they did not have physical education lessons at school or other opportunities to be physically active. Therefore, the co-creation group wanted to organise lunch break activities. The girls immediately emphasised that it would be important to have the support and participation of teachers. The group discussed that they should specifically try to motivate the girls to participate, but that boys could participate as well. During the subsequent co-creation sessions, the intervention became more concrete: it was decided to organise a walk to a park nearby. In the park, participants received a healthy lunch and bottle of water and had the opportunity to walk around in the park or to participate in a sports activity. The group did not want to have a structured sports sessions, but decided to bring some material to the park (e.g., a ball, Frisbee) so that everyone could choose their preferred activity. The co-creation group also chose to give a pedometer to every participant during the walk to motivate them to be active. The girl/boy and teacher with the most steps at the end of the activity received a small incentive. Prior to implementing the intervention, they first wanted to present their idea to some teachers at school. After their approval, the girls presented their intervention to the pupils of the school, using a PowerPoint presentation, to motivate them to subscribe for the lunch walk. After the presentation, pupils and teachers could subscribe for the activity. Due to lack of time, the lunch walk was organised only once and in total, thirteen adolescents participated (including the two girls from the co-creation group). There were also two researchers present during the walk. Of the eleven girls who completed the post-test questionnaire, six (55%) indicated that they participated in the lunch walk. The school coordinator who completed the process evaluation questionnaire was generally very positive about the developed intervention and the co-creation group. Though the school planned to continue with the co-creation group, the school staff lacked time. However, the school submitted a project to get funding to organise a sports activity every month in combination with a healthy lunch, to continue the initiative of the co-creation group. 

### 3.3. Qualitative Process Evaluation of Participating in Co-Creation Group

The most important reasons to participate in the co-creation group were that girls considered it interesting to develop an intervention themselves and that there were, at that time, not many initiatives at school to be active or to do sports. Some girls pointed out that they initially participated because a friend did.

“I thought it was an interesting project.”

*(School 2)*

“We never do physical activities or sports, while most of us would fancy doing something active.”

*(School 3)*

In general, girls were satisfied with the co-creation sessions during lunch break, although some stated that in the future, this might also be organised during lessons.

“It was good to do it during lunch break, we have sufficient time then.”

*(School 2)*

“In the future, it would be better to not have the sessions during lunch break. If we needed an extra 10 minutes to discuss the project, we were not allowed because we have to go to the lesson, so it would be better to do it during school lessons.”

*(School 3)*

They enjoyed the fact that they could give their opinion on possible strategies and that they could develop their own intervention. The girls at school 3 also specifically indicated that they had learned how to present their ideas in front of a group. One girl indicated that she did not have many ideas herself, and that it is therefore positive to have a co-creation group with several girls in order to generate more ideas.

“I really liked it that we could say our own opinion.”

*(School 1)*

“We never have to stand in front of a group, and now we presented the intervention for all those people. A lot of them subscribed for the walk, so we can say that we did a good job!”

*(School 3)*

“It was easier to have a lot of girls in the co-creation group, because then you also have more ideas.”

*(School 2)*

The girls from the co-creation groups indicated that it was positive to include only girls in the co-creation groups, as boys of their age would have had different opinions and ideas about how to promote physical activity, although it was also stated that there could be a co-creation group for boys only as well.

“But I think that if you organise the co-creation group separately for boys, they will like it too!”

*(School 1)*

The girls from the three co-creation groups were satisfied in general with their proposed intervention, but most of them also indicated that the activity could have occurred more often. For school 2, girls indicated that the activity itself did not last long enough. Because the activity only occurred once or too few times, some girls expected that the intervention would not prompt adolescent girls to increase their physical activity levels. However, other girls indicated that the introduction to other sports or activities could incite other girls to do it in their leisure time as well (for example, subscribing to a fitness club). It was also specifically reported in school 3 that you can never satisfy every girl at school with the intervention developed.

“I don’t dance and I am so happy that I got to chance to learn it at school.”

*(School 1)*

“We only had four sport sessions, that is not much.”

*(School 1)*

“Yes, I started with fitness since the activity! I don’t like to do it alone, but since a few weeks, I go to the fitness club with some friends.”

*(School 2)*

“There were more people than I had expected and it was fun, because they all participated in the sports activity in the park, so they did not do it just to be outside of school, so that is really positive.”

*(School 3)*

For the girls, it was important that the school staff approved and supported the intervention because the intervention was located at the school and because they had invested time and effort to co-create it. However, some girls had the feeling that not all teachers were truly supportive of their idea.

“Some people were supportive, but I think all teachers should support our intervention.”

*(School 1)*

“Some teachers tried to encourage us during the fitness activity, but they just sat on a chair themselves.”

*(School 2)*

“Yes, the teachers were supportive, a lot of them also joined the lunch walk and they were really positive about it afterwards.”

*(School 3)*

Specifically for school 1, a negative aspect was that some girls did not want to join the sport sessions because they did not like one or more of the other participating girls. Finally, specifically for school 2, a few girls stated that there is already so much to do or to read on Facebook, that it is difficult to really notice the messages from their own page.

“I don’t like some girls that joined the sport sessions, so I cannot participate, because otherwise they would constantly look at me.”

*(School 1)*

“At this moment, I mostly use Facebook to chat with people, but I don’t scroll anymore to see what has been posted.”

*(School 2)*

Most girls indicated that participating in the co-creation group met their expectations. However, although they were generally satisfied with the developed intervention, some girls indicated that they had expected in advance that the intervention would be more spectacular. One girl admitted that she actually expected that it would be boring to participate in the co-creation group. All girls indicated that they would participate in the co-creation group again if this would be organised in the future. A suggestion was to spend less time with the co-creation group in order to have more time for implementing the intervention.

“I would definitely participate again and I think that next time, more girls would participate!”

*(School 1)*

“Honestly, I thought that we were going to make a more spectacular intervention.”

*(School 2)*

“I would suggest to do less sessions and to do it earlier in the year, so then we have a lot of time left to organise several lunch walks.”

*(School 3)*

### 3.4. Effect Evaluation

Results are presented in Table 5. Two hundred twenty-nine adolescent girls from 27 classes across six schools agreed to participate in the data collection, of which 196 had completed a questionnaire at both pre- and post-test. The analyses included 91 girls from the intervention schools (mean age 15.5 ± 0.6 years) and 105 girls from the control schools (mean age 16.4 ± 1.0 years). There was a significant intervention effect on self-efficacy (*β* = 0.91; standard error (SE) = 0.23; *p* < 0.001): girls from the intervention schools showed an increase in self-efficacy (+0.34 on a 5-point scale), whereas girls’ self-efficacy decreased in the control schools (−0.57 on a 5-point scale). There was also a significant intervention effect on extracurricular sports participation (*β* = 0.73; SE = 0.09; *p* < 0.001): girls from the intervention schools increased their extracurricular sports participation (+0.36 min/day), whereas girls’ extracurricular sports participation decreased in the control schools (−0.11 min/day). No significant intervention effects were found for the other physical activity outcomes, for girls’ perceived barriers and benefits, and for peer and parental modeling of physical activity, co-participation in physical activity and encouragement to be physically active.

## 4. Discussion

The aim of this study was to develop, implement and evaluate a physical activity-promoting intervention in lower-educated adolescent girls using a co-creational approach. Three co-creation groups formed of one researcher and 2 to 13 adolescent school girls designed an intervention specific to their school to change a physical activity behaviour of their choice. In each school, the co-creation group developed a different physical activity-promoting intervention strategy, suggesting that each intervention was tailored to the unique characteristics, needs and interests of the school and its adolescent girls [20,21]. However, the process of setting up a co-creation group and co-creating a school-specific intervention was the same in every school and could therefore be applied in other schools as well [21]. Applying the co-creational approach in other schools would lead to uniquely tailored interventions for adolescent girls. These findings could then be meta-analysed to derive population-level inferences about the effectiveness of such a co-creational approach. This way of scaling the co-created intervention has been described as a distributed model in which local interventions from multiple settings are developed independently and clustered in order to reach a larger population [42]. 

In terms of a process, in this study the co-creation approach was evaluated by the participants using focus groups. The adolescent girls were generally positive about being part of the co-creation group. They all reported they would participate again if a co-creation group would be set up in the future, which might imply that they felt empowered. Also, the inclusion of girls only in the co-creation group was positively received by all girls as they indicated that boys would have launched other ideas or would have taken over the lead. However, it was also reported that installing a co-creation group for adolescent boys would be good. This shows that the girls see value in the process for all, but that they recognise that it needs to be tailored to the specific needs and interest of boys and girls. Adolescents’ interests in and reasons to participate in physical activity indeed differ by gender [43] and physical activity interventions benefit from focusing on girls only [12,13]. In addition, the school staff from the three intervention schools indicated that they liked the concept of establishing a co-creation group and its method of co-creation. These findings suggest that establishing a co-creation group with girls and a researcher at school is a feasible approach to promote physical activity behaviour in adolescent girls with a lower educational level, and that school staff support this way of working. The combination of experience of the target group with their own world and the expertise of the researcher in physical activity promotion in the co-creation group may lead to more acceptable and potentially more effective interventions in this specific target group [44]. It is, however, important that the researcher owns certain skills and competencies such as listening, humility, flexibility and communication (e.g., use of language that is understandable and respectful) [22].

Further, the school staff agreed that it would be possible to set up a co-creation group of adolescent girls themselves. However, no school actually continued with the co-creation group after the project. A possible suggestion for the schools could be to replace the researcher by a teacher or another school staff member. However, it would be unfortunate that the scientific expertise of the researcher is then lacking (i.e., knowledge on the evidence for theory-based, potentially effective intervention strategies). Therefore, it would require a specific phase in which the researcher slowly steps back, thereby transferring all activities and responsibilities to the target group and the school staff involved. In addition, the school staff should then preferably be involved in the co-creation process from the start in order to be suitably trained. A second possibility could be that instead of carrying on with the co-creation group, the schools continue organising the developed interventions after the project. For example, in the Girls in Sport intervention that used a participatory approach, the action team included both school staff and students (and a researcher) in order to share workload and responsibilities and to enhance sustainability if staff or students move from the school [20]. Despite the fact that all three schools indicated that it would be possible to organise the intervention themselves, in our co-creational project the interventions did not continue either. Regardless of which of these two possibilities are applied, both imply the need for more time and effort to embed the approach and/or the interventions in the school culture and procedures.

Although the co-creational approach seems promising and might lead to sustainable solutions for a specific school context, there was still room to optimise the process and each intervention. Generally, all co-created intervention activities could have been organised more often. This was also reported in the focus groups by most girls from the co-creation groups. The preliminary work of co-creating the intervention (brainstorming, developing the idea, presenting the idea to teachers, etc.) took quite a long time, leaving less time to actually implement the intervention within the time frame of a school year. It has indeed been stated that the progress of the entire co-creational process is difficult to predict [22]. The limited intervention intensity or duration could be a possible reason for the lack of effects when evaluating the effect of the co-created physical activity interventions. The results showed that there were generally no statistically significant effects of the co-created interventions, except for time spent in extracurricular sports and girls’ self-efficacy to be physically active. The small effect on extracurricular sports participation is not surprising considering the content of the three interventions developed, but it was insufficient to lead to a detectable increase in the total physical activity level. The moderate increase in self-efficacy might be explained by the fact that girls in interventions schools (and especially in the co-creation group) became more conscious of how physical activity can be promoted or integrated into their daily lives, which resulted in higher beliefs in their capability for being physically active. It has, however, to be kept in mind that the effects were minimal and that effect sizes were small (0.19 for extracurricular sports participation) to moderate (0.63 for self-efficacy). As stated before, more time (and budget) may be needed to truly embed the co-creation group and the interventions developed as part of the school curriculum, in order to induce larger and sustainable effects on physical activity. Another possible reason could be that the co-created interventions were not evaluated with an adequate design. Very few co-created interventions are actually evaluated [17], although these evaluations are needed to find out if co-creation indeed leads to more attractive and effective interventions. Therefore, we considered our effect evaluation of a co-creational approach using a pre-test, post-test design and an intervention and control group as an important study strength. However, the evaluation might not have been designed optimally to capture changes occurring as a result of co-creation. For example, in the current design we considered the intervention group as all girls that were part of one of the three intervention schools in which the co-creational approach was applied, and the control group as all girls from the three control schools. However, there were actually differences among the girls from the intervention group: some were part of the co-creation group, some only participated in the co-created intervention, and others did not participate at all. Unfortunately, the groups were too small to investigate the effect according to these subgroups. Future projects could embed the co-created interventions into a cluster randomised controlled trial and compare them to a traditional top-down intervention to assess their relative effectiveness [42]. In addition, future studies should investigate the effect on other outcomes as well, such as girls’ level of empowerment and feeling of ownership [45], which may in turn lead to behavioural change. 

Other limitations that need to be taken into consideration by future co-creational projects include that some aspects or principles of the co-creational design could have been improved. For example, future projects should try to put more emphasis on shared ownership and girls’ responsibilities, as it was observed that the girls often asked for approval and guidance from the researcher, instead of initiating certain activities themselves. This might be a consequence of the fact that the co-creational process took place at school, so it could be explored if the co-creational process would run differently in a context outside of school (e.g., a neighbourhood community). Also, the girls could have been more involved in other research aspects, such as data collection or dissemination of results, as maximising participation in all research aspects is important [19]. These important issues were not captured via questionnaires or focus groups, but were based on observations made by the researcher, who was part of the co-creation group. As it would be relevant to capture such information in a more structured way, we recommend to use a standardised logbook that could be completed by the researcher (and the target group) after each session. Another limitation is that the co-creation project was planned for one schoolyear, but—as has been repeatedly stated before—more time would be preferable since this approach needs time to fully develop. As a consequence, there was not enough time, for example, to organise the lunch walk more than once because the end of the schoolyear was nearby. In addition, it is important to keep a balance between meeting regularly with the co-creation group and having sufficiently long time between sessions in order to provide enough room to reflect on what has been discussed during the sessions. Since time is such a critical issue, another way of working could be to let the co-creation group pilot and adapt several intervention strategies that have already been developed for (and by) adolescent girls. This way of scaling has been described as a cascade model [42]. However, we hope that this study together with other co-creation studies further inspires health promotion researchers to actively collaborate with their target group when developing interventions in the future [14]. A final study limitation is the use of a questionnaire to assess physical activity because of recall bias and social desirability, although the Flemish Physical Activity Questionnaire has proven to be sufficiently reliable and valid [33]. Objective measurements are recommended, although it has been found that adolescents have the lowest accelerometer-wearing compliance of all age groups [6]. Potentially, when involving the target group in the evaluation aspect of the study (i.e., maximising participation in all research aspects), they could encourage and emphasise the importance of wearing the accelerometer to their peers. As a result, the compliance with these measures might be better.

## 5. Conclusions

In conclusion, actively engaging and involving adolescent girls with a lower educational level during the process of developing and implementing an intervention to promote physical activity seems a feasible approach that deserves further attention in health promotion research. Installing a co-creation group in the school was positively received by both school staff and girls and could be applied to other schools, resulting in school-specific interventions tailored to the needs and interests of the participants. However, there were generally no detectable intervention effects, so future co-creation projects could test this approach more intensively and consider the most optimal co-creation process and evaluation design (including an in-depth process and effect evaluation). Moreover, they should foresee a sufficiently long time period and considerable budget to conduct and embed the co-creational process and/or interventions and consider the involvement of school staff when conducted in the school context.

## Figures and Tables

**Table 1:** Description of individual, sociocultural and school-based variables.

Variable	Questionnaire Item	Item(s) Handled	Response Category
Self-efficacy to be physically active	I could be active even… if I have to get up early if my friends want to do something else if I have a lot of work for school if it is hard or difficult	Mean value of four items (Cronbach’s alpha = 0.85)	1 = Strongly disagree, 2 = Partly disagree, 3 = Neither agree nor disagree, 4 = Partly agree, 5 = Strongly agree
Perceived benefits of physical activity	I think physical activity is good because it improves my aerobic condition and health because I am with friends or I meet new people because I get fun out of physical activities because during physical activities, I can show that I am better than others because otherwise I would feel bored because I lose weight and improve my body	Mean value of six items (Cronbach’s alpha = 0.77)	
Perceived barriers of physical activity	I am not able to participate in physical activities because I do not have enough time because I do not like it because I am not good at it because I do not have transportation to get there because my parents do not allow me	Mean value of five items (Cronbach’s alpha = 0.75)	
Peer modeling of physical activity	How often are your friends physically active?	Single item	1 = Never, 2 = Almost never, 3 = Sometimes, 4 = Often, 5 = Always
Peer co-participation in physical activity	How often are your friends physically active together with you?	Single item	
Peer encouragement to be physically active	How often do your friends encourage you to be physically active?	Single item	
Parental modeling of physical activity	How often are your parents physically active?	Single item	
Parental co-participation in physical activity	How often are your parents physically active together with you?	Single item	
Parental encouragement to be physically active	How often do your parents encourage you to be physically active?	Single item	
Relationship with classmates	My classmates like being together Most of my classmates are friendly and helpful My classmates accept me as I am	Mean value of three items (Cronbach’s alpha = 0.85)	1 = Strongly disagree, 2 = Partly disagree, 3 = Neither agree nor disagree, 4 = Partly agree, 5 = Strongly agree
Involvement in organising school activities	The pupils in our school are involved in organizing school activities	Single item	
Relationship with teachers	My classmates and me have good bonds with the teachers at school	Single item	

**Table 2:** Description of the co-creation group and development and implementation of each school-specific intervention.

Description	School 1	School 2	School 3
General information	Technical and vocational education (Grades 7–12) All girls from one class from Grade 10 were invited to participate in data collection and co-creation group (14 girls in total)	Technical and vocational education (Grades 7–12) All girls from Grade 10 were invited to participate in data collection and co-creation group (8 classes in total; 103 pupils, 89 girls)	Part-time vocational education (≥15 years); the school deals with a lot of absenteeism and drop-out of pupils All girls from the entire school were invited to participate in data collection and co-creation group
Co-creation group	8 girls from one class Mean (standard deviation) age: 15.9 (0.7) years	13 girls from three different classes Mean (standard deviation) age: 16.1 (0.6) years	Initially 4 girls joined the co-creation group, but only 2 girls continued with the co-creation group (1 girl dropped out of school, 1 girl was pregnant and was mostly absent) Mean (standard deviation) age: 17.2 (0.0) years
Developed intervention	4 sport sessions at school during lunch break for girls only 4 different sports that are usually not covered in physical education (Zumba, hiphop dance, kickbox on music, workout with exercises on music) Using flyers and posters to announce the sessions to all girls from Grades 9–12	Facebook page with pictures and movies of exercises to do at home, healthy recipes, tips to be active, etc. One fitness activity for all pupils from Grade 10 (boys and girls), organised during school hours All pupils that took part in the fitness activity, received a bottle of water, an apple and a flyer with the link to the Facebook page	Walk to a park nearby during lunch break with a healthy lunch and the possibility to do a sports activity in the park (e.g., frisbee, soccer); all pupils (boys and girls) were invited to join and all participating pupils were invited to wear a pedometer during the activity to record the amount of steps The pupil with the most steps during the walk received an incentive Developing a teacher manual with tips to increase the activity levels of pupils PowerPoint presentation to all pupils and teachers of the school to encourage them to participate
Implementation of the intervention	Session 1 (Zumba): 9 participants (including 4 girls from the co-creation group and 5 girls from other classes/grades) Session 2 (Hiphop dance): 11 participants (including 3 girls from the co-creation group and 8 girls from other classes/grades) Session 3 (Kickbox on music): 11 participants (including 3 girls from the co-creation group and 8 girls from other classes/grades) Session 4 (Workout): 14 participants (including 3 girls from the co-creation group and 11 girls from other classes/grades)	Grade 10 was divided into 4 groups; 2 groups each went to a fitness center during the morning school hours and 2 groups each went to a fitness center during the afternoon school hours Every group was supervised by at least two school teachers All pupils from Grade 10 were subscribed to the fitness activity (103); but there are no official records how many pupils were actually present Both the girls and the researcher had access to post something on the Facebook page, but most posts were placed by the researcher and only few posts were placed by the girls	13 pupils participated in the lunch walk (including the 2 girls from the co-creation group) One of the girls from the co-creation group had the highest amount of steps (6005 steps) and received a small incentive

**Table 3:** Process evaluation of the three interventions based on girls’ questionnaire data.

School 1	School 2	School 3
13 girls completed the post-test questionnaire. Only 3 girls from the co-creation group/selected class participated in the 4 sport sessions and completed the process evaluation questions. Those 3 girls were asked to what degree they agreed with five statements: 1 “I liked the sport sessions” (2 girls totally agreed, 1 girl somewhat agreed) 2 “I liked the fact that the sport sessions were for girls only” (2 girls totally agreed, 1 girl was neutral) 3 “The offered sports were fun” (2 girls totally agreed, 1 girl was neutral) 4 “I would like it if the sport sessions would be organised again in the future” (2 girls totally agreed, 1 girl was neutral)	62 girls completed the post-test questionnaire, of which 49 indicated that they participated in the organised fitness activity (79%). Those 49 girls were asked to what degree they agreed with four different statements: 1 “I enjoyed taking part in the fitness activity” (68% totally agreed; 22% somewhat agreed, 6% was neutral, 2% somewhat disagreed, 2% totally disagreed) 2 “Fitness is a fun activity, as everyone can chose what he/she does at the fitness” (86% totally agreed; 10% somewhat agreed, 4% was neutral) 3 “I would like it if the fitness activity would be organised again in the future” (78% totally agreed; 16% somewhat agreed, 4% somewhat disagreed, 2% totally disagreed) 4 “The fitness activity has prompted me to be more active” (36% totally agreed; 24% somewhat agreed, 18% was neutral, 6% somewhat disagreed, 16% totally disagreed) 32% (20/62) indicated that they ‘liked’ the page on Facebook. The most important reasons not to ‘like’ the Facebook page, were that they did not know about this page and that they do not have a Facebook account. Those 20 students were asked to what degree they agreed with four different statements: 1 “I think the Facebook page is fun” (30% totally agreed, 30% somewhat agreed, 25% was neutral, 10% somewhat disagreed, 5% totally disagreed) 2 “The posts on the Facebook page are interesting” (35% totally agreed, 15% somewhat agreed, 35% was neutral, 10% somewhat disagreed, 5% totally disagreed) 3 “The posts on the Facebook page encourage me to be more active or to eat more healthy” (15% totally agreed, 20% somewhat agreed, 45% was neutral, 10% somewhat disagreed, 10% totally disagreed) 4 “I think that there should be more posts on the Facebook page” (40% totally agreed, 40% somewhat agreed, 15% was neutral, 5% totally disagreed)	11 girls completed the post-test questionnaire, of which 6 girls (55%) indicated that they participated in the lunch walk. The most important reasons not to participate in the lunch walk were that they were absent that day, that they did not have time to do a lunch walk, or that they do not like walking. Those 6 girls were asked to what degree they agreed with three different statements: 1 “I enjoyed taking part in the lunch walk to the park” (5 girls totally agreed, 1 girl somewhat agreed) 2 “I enjoyed taking part in the sports activity at the park” (3 girls totally agreed, 2 girls somewhat agreed, 1 girl was neutral) 3 “I would like it if the lunch walk was organised again the future” (4 girls totally agreed, 2 girls were neutral)

**Table 4:** Process evaluation of the three interventions based on school staff questionnaire data.

Process Evaluation Questions	School 1	School 2	School 3
It was good that the co-creation group could think of their own intervention to promote physical activity	Totally agree	Totally agree	Totally agree
Setting up a co-creation group with pupils fitted within the school’s view	Somewhat agree	Totally agree	Totally agree
The applied co-creational approach was good (i.e., a co-creation group of adolescents and a researcher during lunch break)	Totally agree	Totally agree	Totally agree
It was good that the co-creation group only consisted of girls	Neutral	Somewhat agree	Somewhat agree
It would be possible for the school to set up such co-creation group	Somewhat agree	Somewhat agree	Totally agree
The school has set up such co-creation group this school year	Totally disagree	Totally disagree	Neutral
I was satisfied with the developed intervention of the co-creation group	Somewhat agree	Somewhat agree	Totally agree
The intervention was in line with my expectations	Somewhat agree	Totally agree	Totally agree
The intervention promoted the adolescent girls to be more active	Totally agree	Somewhat agree	Totally agree
The intervention fitted within the school’s view	Totally agree	Totally agree	Totally agree
It would be possible for the school to organise such intervention activities	Totally agree	Somewhat agree	Totally agree
The school has organised such intervention activities this school year	Totally disagree	Somewhat disagree	Neutral

**Table 5:** Intervention effect on physical activity outcomes, individual, sociocultural and school-based factors.

Variable (Scale)	Mean Value at Pre-Test	Mean Value at Post-Test	Interaction Effect Time * Group *β* (Standard Error)
Total physical activity (min/day)	I: 52.22	I: 49.34	−4.44 (4.06)
	C: 56.67	C: 58.23	
Active transportation school (min/day)	I: 1.17	I: 0.68	−0.85 (2.25)
	C: 3.53	C: 4.23	
Active transportation leisure time (min/day)	I: 40.60	I: 37.51	−5.49 (3.30)
	C: 30.78	C: 33.18	
Extracurricular sports (min/day)	I: 0.01	I: 0.37	0.73 (0.01) *
	C: 0.47	C: 0.36	
Sports participation leisure time (min/day)	I: 16.35	I: 15.64	0.01 (0.07)
	C: 23.40	C: 22.65	
Self-efficacy (1–5)	I: 2.81	I: 3.15	0.91 (0.23) *
	C: 3.30	C: 2.73	
Perceived benefits (1–5)	I: 3.60	I: 3.54	−0.10 (0.10)
	C: 3.72	C: 3.76	
Perceived barriers (1–5)	I: 2.58	I: 2.57	−0.03 (0.12)
	C: 2.45	C: 2.47	
Peer modeling (1–5)	I: 2.67	I: 2.81	0.07 (0.16)
	C: 3.14	C: 3.21	
Parental modeling (1–5)	I: 2.63	I: 2.62	−0.11 (0.16)
	C: 2.50	C: 2.59	
Peer co-participation (1–5)	I: 2.33	I: 2.44	0.27 (0.17)
	C: 2.62	C: 2.46	
Parental co-participation (1–5)	I: 1.86	I: 1.81	−0.28 (0.15)
	C: 1.73	C: 1.96	
Peer encouragement (1–5)	I: 2.20	I: 2.28	0.02 (0.21)
	C: 2.38	C: 2.43	
Parental encouragement (1–5)	I: 2.66	I: 2.91	0.34 (0.22)
	C: 3.03	C: 2.94	
Relationship classmates (1–5)	I: 4.05	I: 3.79	−0.08 (0.19)
	C: 4.46	C: 4.28	
Involvement in school activities (1–5)	I: 3.04	I: 3.03	0.13 (0.23)
	C: 3.54	C: 3.39	
Relationship teachers (1–5)	I: 3.46	I: 3.21	−0.30 (0.18)
	C: 3.17	C: 3.21	

* *p* < 0.001.

## Supplementary Materials

The following are available online at www.mdpi.com/1660-4601/14/8/862/s1.
